# Skeletal involvement in the pathogenesis and outcomes of rheumatoid arthritis and osteoarthritis

**DOI:** 10.1186/ar3571

**Published:** 2012-02-09

**Authors:** Jean-Pierre Pelletier

**Affiliations:** 1 University of Montreal and University of Montreal Hospital Centre (CHUM), Montreal, Quebec, Canada

## 

Bone remodeling is a frequently observed phenomenon in musculoskeletal diseases such as rheumatoid arthritis (RA) and osteoarthritis (OA). The level of imbalance between bone resorption/deposition is responsible for the morphological changes osteopenia/bone erosion/osteosclerosis observed in these arthritic conditions.

In RA, increased osteoclastic activity is responsible for the development of focal osteopenia/erosion and systemic osteoporosis. The increased osteoclast activity in RA has been demonstrated to be linked to a dysregulation of pathways including cell-cell interactions, cytokines, and the receptor activator of nuclear factor κB (RANK)/RANK ligand (RANKL) system. Recent studies have shown that joint erosion in RA is linked to a decrease in long-term physical function.

Under OA conditions, the subchondral bone is the site of numerous dynamic morphological changes. These changes are associated with a number of local abnormal biochemical pathways related to the altered metabolism of osteoblasts and osteoclasts. At the early stages of the disease process, increased bone loss and resorption is observed with subchondral bone associated with local production of catabolic factors including cathepsin K and MMP-13. Moreover, OA osteoblasts present an abnormal phenotype resulting in increased production of growth hormones and catabolic factors. In addition, factors such as osteoprotegerin (OPG) and RANKL have been found to be expressed and modulated over time in human OA subchondral bone. Their synthesis varies from being reduced in early OA to being increased in the late stages of the disease. This finding may explain that in the early stages of OA, bone remodeling favors resorption and in the more advanced stages of the disease, bone formation is predominant.

Magnetic resonance imaging (MRI) studies in knee OA patients have shown that the subchondral bone is frequently the site of signal alterations-bone marrow lesions (BML) - indicative of a great variety of morphological changes. BML and cartilage loss have been linked in several studies. Moreover, studies have identified, in OA patients, a number of risk factors for total knee replacement including BMLs.

The paradigms regarding the role of bone lesions in arthritic diseases raise a number of important questions. A comprehensive understanding of the factors that contribute to these changes will provide us with better knowledge of the pathophysiology of the diseases and the role of these structural alterations in patient symptoms and prognosis, as well as guiding the development of new therapeutic strategies.

